# Free ISG15 triggers an antitumor immune response against breast cancer: a new perspective

**DOI:** 10.18632/oncotarget.3372

**Published:** 2015-01-31

**Authors:** Julian Burks, Ryan E. Reed, Shyamal D. Desai

**Affiliations:** ^1^ Department of Biochemistry & Molecular Biology, LSU Health Sciences Center-School of Medicine, New Orleans, LA, USA; ^2^ Present Address: Georgetown University Medical Center, Lombardi Comprehensive Cancer Center Department of Molecular Oncology, Washington, DC, USA

**Keywords:** ISG15, Ubiquitin/proteasome, Antitumor, Immune system, Breast cancer

## Abstract

Interferon-Stimulated Gene 15 (ISG15), an antagonist of the canonical ubiquitin pathway, is frequently overexpressed in various cancers. In cancer cells, ISG15 is detected as free (intracellular) and conjugated to cellular proteins (ISGylation). Free ISG15 is also secreted into the extracellular milieu. ISGylation has protumor functions and extracellular free ISG15 has immunomodulatory properties *in vitro*. Therefore, whether ISG15 is a tumor suppressor or tumor promoter *in vivo* remains controversial. The current study aimed to clarify the role of free ISG15 in tumorigenesis. Breast cancer cells stably expressing control, ISG15, and UbcH8 (ISG15-specific E2 ligase) shRNAs were used to assess the immunoregulatory and antitumor function of free ISG15 in cell culture (*in vitro*) and in nude mice (*in vivo*). We show that extracellular free ISG15 suppresses breast tumor growth and increases NK cell infiltration into xenografted breast tumors in nude mice, and intracellular free ISG15 enhances major histocompatibility complex (MHC) class I surface expression in breast cancer cells. We conclude that free ISG15 may have antitumor and immunoregulatory function *in vivo*. These findings provides the basis for developing strategies to increase systemic levels of free ISG15 to treat cancer patients overexpressing the ISG15 pathway.

## INTRODUCTION

Interferon-Stimulated Gene 15 (ISG15) has emerged as a promising and novel oncoprotein biomarker elevated in various cancers [1-3]. Although the role of ISG15 in host defenses against bacterial and viral pathogens is well documented [4-6], very little is known about its role in cancer. Currently, the literature suggest that ISG15 has both antitumor and protumor properties, reviewed in [2]. However, whether ISG15 promotes or suppresses tumor growth *in vivo* remains controversial.

ISG15 was the first ubiquitin-like protein identified and is comprised of two ubiquitin-like β-grasp domains connected by a linker region [7]. The presence of a canonical ubiquitin C-terminal LRLRGG sequence very early suggested that the polypeptide exerted its biological effects through covalent conjugation to cellular protein targets [7], later confirmed by Western blot [8] and immunohistochemistry [9]. In parallel with ubiquitin and similar pathways, ISG15 conjugation (ISGylation) requires three distinct enzymes: an ATP-dependent activating enzyme for ISG15 (UbE1L), several ISG15-specific conjugating enzymes (Herc5 and EFP, among others) that append the activated ISG15 to specific cellular target proteins, and an ISG15-specific carrier protein/conjugating enzyme (UbcH8) that functionally connects the activation and conjugation half reactions [10, 11]. Thus, ISG15 exists in both free and conjugated pools within cells, both of which are often elevated in cancer, although the basis for differences in cellular levels among different tumors remains unclear [12].

Recent studies from our group revealed that ISG15 inhibits polyubiquitylation, consequently inhibiting subsequent degradation of specific cellular proteins in breast cancer cells [12-15]. We have demonstrated that ISG15 stabilizes key cellular proteins involved in cell migration/metastasis, conferring increased motility to breast cancer cells (13) and promotes breast cell transformation [13, 14]. Remarkably, ablating ISG15 conjugation by blocking expression of ISG15 or UbcH8 reverses the transformed phenotype [11, 12]. Others have subsequently demonstrated that enhanced ISGylation promotes prostate cancer cell transformation [15]. Thus, these results revealed that ISG15 conjugation (ISGylation) has a protumor function, presumably by disrupting normal cellular protein homeostasis mediated through the Ubiquitin Proteasome Pathway.

The ISG15 polypeptide is also secreted from cells through a noncanonical pathway into the extracellular milieu where it functions as an immunomodulatory cytokine [16, 17]. Previous work demonstrated that extracellular free ISG15 can activate natural killer (NK) cells (18), induce non-major histocompatibility complex-restricted cytolysis of tumor cell targets by NK-derived lymphokine-activated killer (LAK) cells [18], stimulate IFN*γ* production from T cells [18], induce dendritic cell maturation [19], and neutrophil recruitment [19]. These studies suggest that free extracellular ISG15 has antitumor properties.

In the current study, we have sought to clarify the role of cellular and extracellular free ISG15 in tumorigenesis using nude mice and cell culture models. We provide evidence that ISG15-silenced tumors grow rapidly compared to ISG15 overexpressing tumors in nude mice, that recombinant free ISG15 inhibits tumor growth when added extracellularly and induce intratumor infiltration of NK cells in nude mice, and that intracellular free ISG15 enhances 26S proteasome-dependent surface expression of MHC class I complexes on breast cancer cells. Together, our results reveal that free ISG15 exerts an antitumor effect by activating the innate and adaptive arms of the immune system *in vivo*.

## RESULTS

### ISG15 inhibits breast tumor growth in nude mice

**Figure 1:**
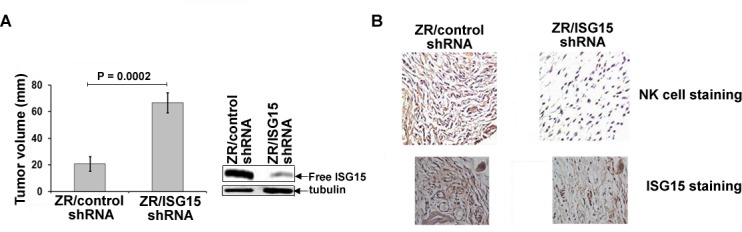
ISG15 inhibits ZR-75-1 breast tumor growth in nude mice and induces NK cell filtration into xenograft tumors A, ZR/control and ISG15 shRNA xenografts were established in female nude mice, and tumor volume was measured two weeks after implantation as indicated in Methods. Each bar represents average volume calculated from eight mice (four mice/group in two different experiments) (Bars: +/− SEM). B, Frozen tumor sections (from A) were immunostained using anti-CD49b-DX5 antibodies (upper panels) or anti-ISG15 antibodies (lower panels) as described in Methods.

To test whether ISG15 contributes to tumorigenesis *in vivo*, we examined the ability of the ZR-75-1 breast cancer cells constitutively overexpressing ISG15 (ZR/control shRNA) and ZR-75-1 cells silenced for ISG15 expression (ZR/ISG15 shRNA), described in [13, 20], to form tumors in nude mice (Ncr^nu/nu^, Jackson Laboratory). Because constitutive ISG15 induction confers a malignant phenotype on breast cancer cells [13, 14], we anticipated that ZR/control shRNA cells overexpressing ISG15 would form large tumors compared to the ZR/ISG15 shRNA cells silenced for the ISG15 expression. Remarkably, we found that ZR/control shRNA cells formed small tumors while ZR/ISG15 shRNA cells formed large tumors in nude mice in the same time-frame (Figure 1A). The average size of the ISG15 overexpressing tumors (20 mm) was significantly smaller than that of the ISG15-silenced tumors (70 mm) (P = 0.0002). Western blot analysis of pooled lysates confirmed significant ablation of free ISG15 pools in ZR/ISG15 shRNA cultures compared to ZR/control shRNA cells (Figure 1A). These results suggest that free ISG15 in part suppresses tumor growth *in vivo*.

### ISG15 promotes intratumor infiltration of NK cells

Because recombinant ISG15 is known to stimulate the proliferation and activation of NK cells in culture [18], we examined the possibility of ISG15 secretion from the tumor cells and activation of NK cells as a plausible reason for the regression of ISG15 overexpressing tumors in nude mice. We evaluated the extent of NK cell infiltration in ZR/LV-control and -ISG15 shRNA tumor sections by immunohistochemical analysis using the anti-NK cell-specific antibody DX5 (anti-CD49b-DX5 clone) [21]. The same xenograft tumors shown in the Figure 1A were used for immunohistochemistry. As shown in Figure 1B, a significant increase in anti-NK cell positive staining (brown color) was found in ZR/control shRNA xenograft tumor sections (Figure 1B, left panel) but no apparent NK cell staining in ZR/ISG15 shRNA xenograft tumors (Figure 1B, right panel), ruling out cross-reaction of the anti-NK cell-specific immunoprobe with the tumor sections. The same tumor sections were also stained in parallel with murine ISG15-specific antibodies, which demonstrated significantly ablated ISG15 signal in ZR/ISG15 shRNA compared to ZR/control shRNA xenografts (panel B). These results together with the tumor regression results shown in Figure 1A are consistent with ISG15-dependent NK cell induction and infiltration into the xenograft tumors leading to regression of tumor growth.

### ISG15-mediated inhibition of tumor growth and NK cells infiltration are general phenomena

**Figure 2:**
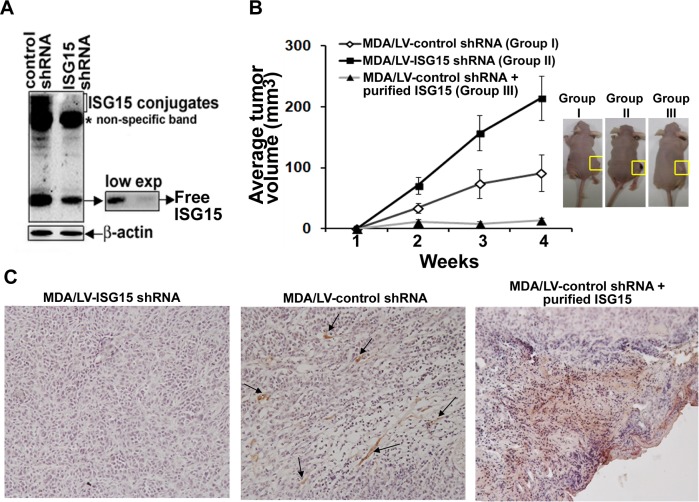
ISG15 inhibits MDA-MB-231 breast tumor growth in nude mice and induces NK cell filtration into xenograft tumors A, ISG15 expression in MDA/LV-control and ISG15 shRNA was analyzed using anti-ISG15 antibodies in Western analysis as described in Methods. B, MDA/LV-control (Group I) and ISG15 (Group II) shRNA xenografts were established in female nude mice. On the same day, recombinant free ISG15 (188 ug) (Boston Biochemical) was injected near the site of xenograft implantation in Group III. Tumor volume was measured from 0-3 weeks after implantation as indicated in Methods. Each point represents average volume (+/− SEM) calculated from eight different tumors for Group I and II, and four different tumors for Group III. Representative photographs of tumor-bearing mice after three weeks of all test groups are shown in the right panel. C, Frozen tumor sections (from Group I, II, and III shown in A) were immunostained using anti-CD49b-HHMα2 antibodies as described in Methods.

To test the generality of our results, we examined the ability of MDA-MB-231 clonal breast cancer cells overexpressing lentiviral control (MDA/LV-control shRNA) or ISG15 (MDA/LV-ISG15 shRNA) shRNAs (described in [14]) to form tumors in nude mice (Ncr nude) (Taconic Company). The Western blot of Figure 2A demonstrates the efficient knockdown of free ISG15 expression in MDA/LV-ISG15 shRNA cells, emphasized at lower exposure (side panel). Similar to ZR/control shRNA cells (Figure 1A), MDA/control shRNA xenografts tumors constitutively overexpressing ISG15 grew slower in nude mice (Figure 2B, closed squares). On the other hand, MDA/LV-ISG15 shRNA tumor xenografts grew rapidly in nude mice (Figure 2B, open squares). The average size of the ISG15 overexpressing tumors (66 mm in 3 weeks) was significantly lower than the ISG15-silenced tumors, measured three weeks post-implantation of tumor cells into nude mice (126 mm in 3 weeks) (P = 0.033). Pictures of tumor bearing mice are shown in Figure 2B, right panel. These results, together with the results obtained using ZR-75-1 breast cancer cells (shown in Figure 1), revealed that ISG15 in part suppresses tumor growth *in vivo*.

We also examined MDA-MB-231 tumor xenografts for NK cell infiltration, this time using anti-CD49b-HMα2 antibody in immunohistochemical analysis. As shown in Figure 2C, first panel, no apparent NK cell positive staining was observed in MDA/LV-ISG15 shRNA xenograft sections. On the other hand, a significant amount of NK cell positive staining was observed in MDA/LV-control shRNA tumor xenograft tissue sections (overexpressing ISG15), suggestive of the increased NK cell infiltration into these tumors (Figure 2C, second panel, arrows). Tumor section staining with IgG as control did not show any staining, demonstrating the specificity of anti-NK cell antibodies used in our analysis (data not shown). Together, results using two different antibodies directed against CD49b, and two different breast cancer xenografts implanted in two different colonies of nude mice demonstrate that ISG15 induces infiltration of NK cells, and inhibits tumor growth in nude mice.

### Extracellular free ISG15 induces intratumoral infiltration of NK cells and inhibits tumor growth in nude mice

Free ISG15 is reported to serve as a chemotactic factor for neutrophils [19]. We therefore tested whether addition of purified recombinant free ISG15 extracellularly was capable of stimulating chemotaxis and migration of NK cells into tumors to inhibit tumor growth in nude mice as a means of simulating secretion of the polypeptide. The ISG15 pathway is overexpressed in most human malignancies. Hence, to make our study clinically relevant, ISG15 overexpressing MDA/LV-control shRNA cells were xenografted into nude mice. On the same day, recombinant purified free ISG15 (188 μg/animal; Boston Biochemical) was injected subcutaneously near the site of tumor implantation. Interestingly, we observed almost complete suppression of the MDA/LV-control xenograft tumor growth in nude mice injected with free ISG15 (Figure 2B, closed triangles). We also found increased NK cell staining in MDA/LV-control xenografted tumor sections (overexpressing ISG15) in mice administered with free ISG15 (Figure 2C, third panel). A ToxinSensor^TM^ Chromogenic LAL Endotoxin Assay (GenScript) of the ISG15 preparation failed to detect LPS (see Material and Methods). Also, commercial ISG15 used in this study failed to induce the ISG15 pathway in RAW 264.7 mouse macrophage cells (data not shown). These two experiments confirmed that the observed antitumor effect and NK cell migration was solely due to the free ISG15 and not potential contamination from any LPS in the commercial ISG15 preparation. Together, our results suggest that extracellular free ISG15 *stimulates infiltration of NK cells,* which in turn, may lead to suppression of tumor growth in nude mice.

### Intracellular free ISG15 enhances antigen presentation in breast cancer cells

**Figure 3:**
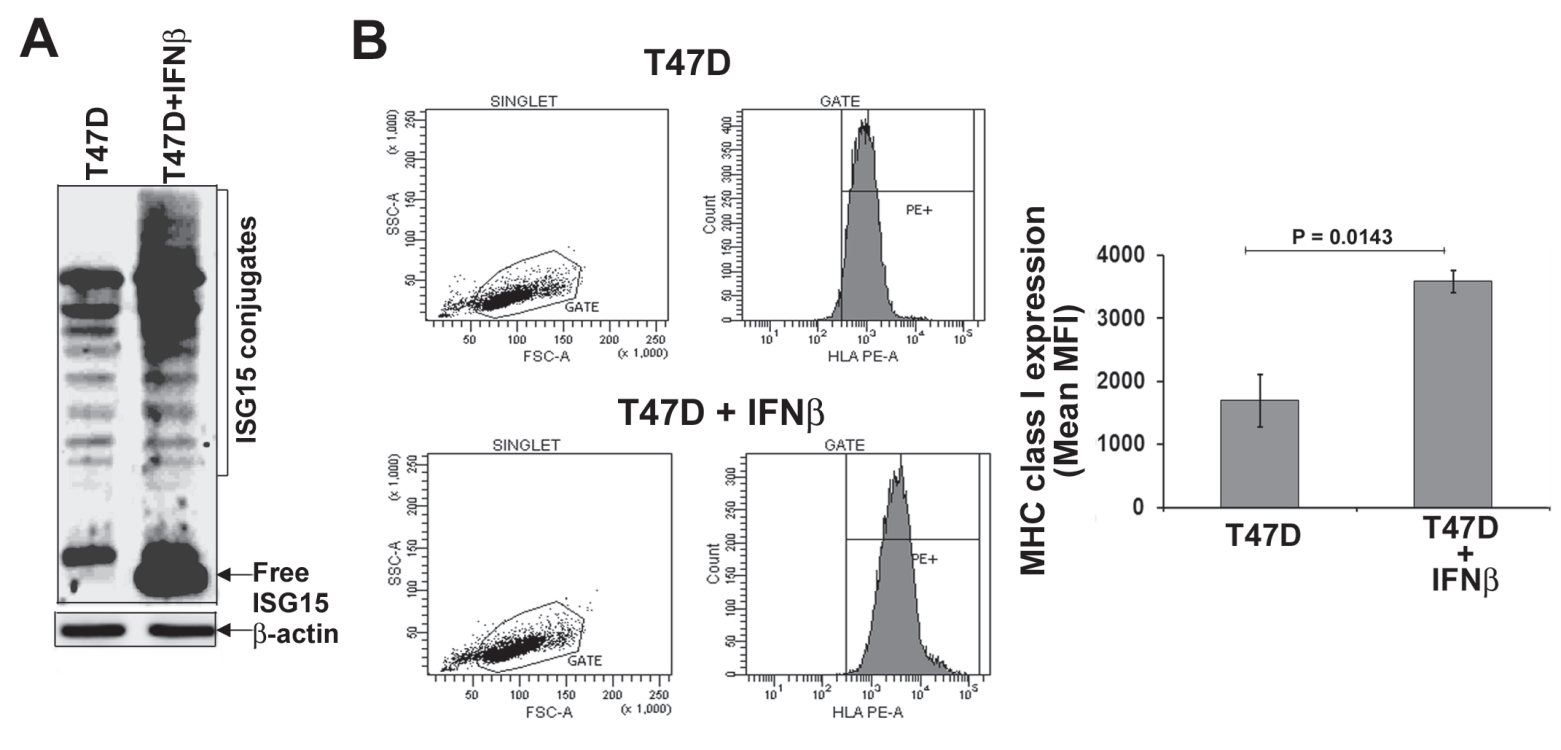
IFNβ increases MHC class I surface expression in T47D breast cancer cells A, T47D breast cancer cells were left untreated or treated with human IFNβ (1000 units/ml) for 24 hrs. Cell lysis and immunoblotting analysis using anti-ISG15 antibodies was carried out as described in Methods. B. Flow cytometric analysis of MHC class I (HLA class I ABC-PE) surface expression on T47D and IFNβ-treated T47D breast cancer cells (from A) is shown. Mean values of the median fluorescence intensity from three independent experiments are plotted in the bar graph (Bars: +/− SEM).

ISG15 is a potential tumor antigen [22]. The effective antigen presentation by MHC class I molecules is essential to activate the adaptive arm (T cell activation) of the immune system [23]. To test the potential role of ISG15 in activating the adaptive arm of the immune system, we assessed MHC class I surface expression, a marker for efficient antigen presentation, on T47D breast cancer cells devoid of free ISG15 expression and IFNβ-treated T47D cells expressing high levels of ISG15. Figure 3A shows that the ISG15 pathway is induced in the IFNβ-treated T47D cells. The same cells were used for assessing MHC class I surface expression. The MHC class I surface expression was assessed by flow cytometry analysis using an anti-human HLA-ABC PE antibody. As shown in Figure 3B, IFNβ-treated T47D cells overexpressing the ISG15 pathway displayed a 2-fold increase in levels of surface MHC class I expression (lower panels) compared to untreated T47D cells (upper panels). The experiment was independently repeated three times and the mean values of the median MHC class I fluorescence intensity are plotted in the accompanying bar graph (Figure 3B, right panel). Increased MHC class I surface expression suggested that IFNβ, a major inducer of the ISG15 pathway, promotes antigen presentation. This study corroborates the literature that elevated IFNβ signaling stimulates MHC class I expression in breast cancer cells [24] and other studies that IFNs induces MHC class I surface expression on cancer cells [25,26].

**Figure 4:**
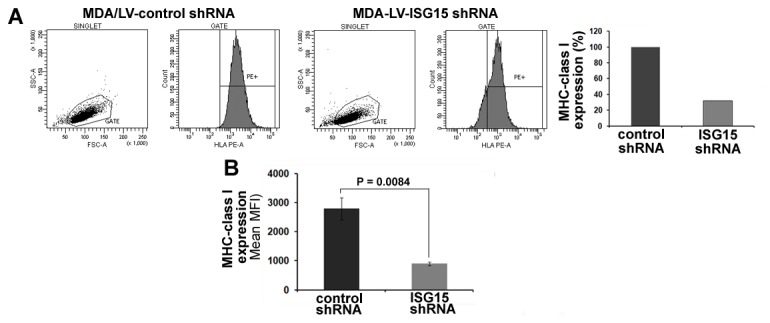
MHC class I surface expression is increased in ISG15 overexpressing breast cancer cells A, A representative flow cytometric analysis of MHC class I surface expression on MDA/LV-control and ISG15 shRNA cells is shown. Bar graph shows the quantification of the flow cytometric data shown in A. B, Experiment shown in panel A was repeated three times and the mean values of the median fluorescence intensity are plotted in the bar graph (Bars: +/− SEM).

Interferonβ stimulates the expression of >1000 genes, collectively called as ISGs (Interferon Stimulated Genes), including ISG15 [27-29]. Hence, it was not clear if the increased surface expression of MHC class I is exclusively due to the elevated expression of ISG15 in the IFNβ-treated T47D cells. We therefore assessed *MHC* class I surface expression on the MDA/LV-control shRNA breast cancer cells constitutively overexpressing ISG15 and MDA/LV-ISG15 shRNA cells silenced for ISG15 expression by the flow cytometry. Similar to the IFNβ-treated T47D cells, ISG15 overexpressing MDA/LV-control shRNA cells displayed 3-fold more MHC class I on their surfaces compared to the ISG15-silenced MDA/LV-ISG15 shRNA cells (Figure 4A, first and second panels, and the bar graph in the third panel for quantitation). The experiment was independently repeated three times and the mean values of the median MHC class I fluorescence intensity are plotted in the bar graph (Figure 4B). Similar to MDA/LV-ISG15 shRNA cells, ZR/ISG15 shRNA also showed increased MHC class I surface expression compared to ZR/control shRNA cells (data not shown). Together, results using IFNβ-treated T47D overexpressing ISG15, MDA/LV-control shRNA constitutively overexpressing ISG15, and ZR/control shRNA breast cancer cells constitutively overexpressing ISG15 reveal that ISG15 increases MHC class I surface expression and antigen presentation in breast cancer cells.

### Increased MHC class I surface expression is dependent on the function of 26S proteasome in breast cancer cells

To test whether the increased surface expression of MHC class I observed in MDA/LV-control shRNA cells is dependent on the function of 26S proteasome to cleave intracellular proteins for surface presentation, we assessed MHC class I surface expression by flow cytometry on MDA/LV-control shRNA cells in the absence or presence of the MG132 proteasome inhibitor.

**Figure 5:**
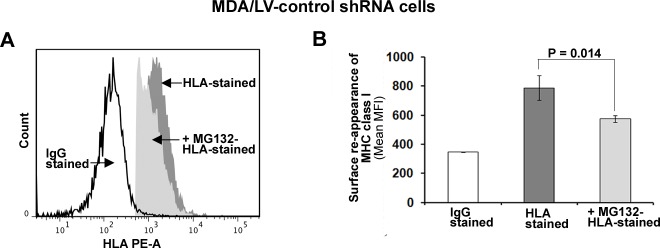
MHC class I surface expression is dependent on the function of 26S proteasome in ISG15 overexpressing breast cancer cells A, Cell surface MHC class I molecules on MDA/LV-control shRNA cells were removed by acid stripping for 90s. Reappearance of MHC class I surface expression was then measured in the presence/absence of MG132 (5 μM) after 6 hr by flow cytometric analysis as described in Figure 4. A representative flow cytometric graph for MHC class I surface reappearance on MDA/LV-control cells in the presence/absence of MG132 is shown. B, Experiment shown in panel A was repeated three times and the mean values of the median fluorescence intensity are plotted in the bar graph (Bars: +/− SEM).

We first treated cells with MG132 for 6h and then assessed MHC class I surface expression in MDA/LV-control shRNA cells using flow cytometry as described above. Under these conditions, no difference in MHC class I surface expression in the absence or presence of MG132 was observed (not shown). Cells were then treated with acid to “strip-off” existing MHC class I complexes from the cell surfaces as described previously [30]. Acid-stripped cells were then cultured in the absence/presence of 5 μM MG132 for 6 hrs at 37^0^C. The re-expression of MHC class I on MDA/LV-control/ISG15 shRNA cells was then assessed using flow cytometry as described in Figure 4. As shown in Figure 5A, MG132 treatment blocked re-expression of MHC class I by 37-40% in acid-stripped MDA/LV-control shRNA cells. Mean values of the median MHC class I fluorescence intensity from three experiments are plotted in the bar graph of Figure 5B. Involvement of the proteasome pathway in MHC-class I antigen presentation has been well documented previously [31, 32]. However, recent studies have revealed a role of the autophagy pathway in MHC class I-mediated antigen presentation [33]. Our results that MG132 blocks MHC-class I presentation thus suggest that increased MHC class I presentation in ISG15 overexpressing breast cancer cells is dependent on proteasome function but not the autophagy pathway.

### Free ISG15 but not ISGylation enhances MHC class I presentation in breast cancer cells

**Figure 6:**
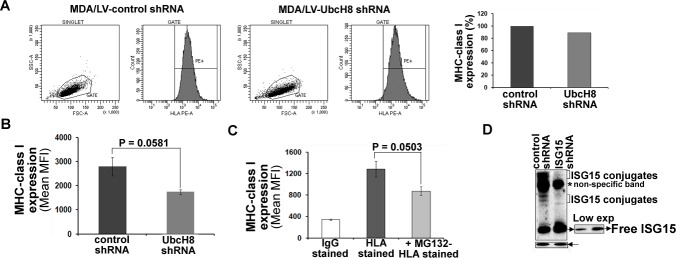
Free ISG15 and not ISGylation contributes to increased MHC class I surface expression in breast cancer cells A, A representative graph of flow cytometric analysis of MHC class I surface expression on MDA/LV-control and UbcH8 shRNA cells is shown. Bar graph shows the quantification of the flow cytometric data shown in A. B, Experiment shown in panel A was repeated three times and the mean values of the median fluorescence intensity are plotted in the bar graph. B) Reappearance of MHC class I surface expression was measured (three experiments) in the presence/absence of MG132. C, Expression of ISG15 and conjugates using anti-ISG15 antibodies is shown. All bars: +/− SEM.

Both free and conjugated forms of ISG15 are induced in IFNβ-treated T47D (Figure 3A) and MDA/LV-control shRNA (Figure 1A) breast cancer cells. Hence, it was not clear whether free ISG15 or the subsequent conjugation of the polypeptide to cellular targets promotes enhanced MHC class I presentation. To resolve this question of the provenance of MHC class I surface expression, we employed the UbcH8-silenced MDA-MB-231 (MDA/LV-UbcH8 shRNA) cell line previously reported [14]. The UbcH8 polypeptide is an E2 conjugating enzyme/carrier proteins essential for ISG15 conjugation to cellular proteins (34,35). Therefore, MDA/LV-UbcH8 shRNA cells have free ISG15 but are blocked from subsequent conjugation by the absence of the required cofactor for the conjugation pathway (Figure 6D). Interestingly, MDA/LV-UbcH8 shRNA cells displayed MHC class I on their surface to almost the same extent as MDA/LV-control shRNA cells (Figure 6A and the accompanying bar graph for quantitation). Mean MHC class I fluorescence intensity values of three different experiments revealed that MHC class I expression in MDA/LV-UbcH8 cells was 62% of that observed in MDA/LV-control shRNA cells (Figure 6B). MHC class I expression was also found to be proteasome dependent in MDA/LV-UbcH8 cells (Figure 6C). Notably, MHC class I expression in MDA/LV-ISG15 shRNA was only 30% (in three different experiments) of that observed in MDA/LV-control shRNA cells (compare Figure 4B and Figure 6B). Since MDA/LV-UbcH8 cells have free ISG15 but lack ISG15 conjugates (Figure 6D), 30% more surface expression in UbcH8 shRNA cells suggest that free ISG15 rather than ISG conjugation to intracellular targets is required for increased antigen presentation in breast cancer cells.

## DISCUSSION

*In vitro* cell culture studies have identified ISG15 as a “double-edged sword protein” with both antitumor and protumor functions. While initial studies revealed that free ISG15 has antitumor function [18, 19, 36], there is a growing evidence supporting that ISGylation (ISG15 conjugates) has protumor function *in vitro* [13-15]. Therefore, whether the ISG15 pathway which is highly elevated in most cancers, is a friend or foe during tumorigenesis *in vivo* is unclear. Current study was undertaken to test the role of ISG15 in tumorigenesis finding that free ISG15 has antitumor function *in vivo*. We show that ectopic injection of free ISG15 (extracellular free ISG15) suppresses growth of the ISG15 pathway overexpressing xenografted tumors *in vivo*, and enhances NK cell infiltration into tumors. Earlier studies have revealed that free ISG15 induces IFNγ producing CD56^+^ NK cell population suggesting the role of secreted ISG15 in establishing IFNγ-mediated antitumor innate response [18]. Further studies using anti-CD11b antibodies (equivalent of CD56+ in human [37, 38]) are needed to examine whether ISG15-mediated increased infiltration of NK cells observed in our study is indeed for establishing IFNγ-mediated antitumor innate immune response in nude mice.

How ISG15 enhances NK cell infiltration has not been studied. However, free ISG15 is a *chemotactic* factor for neutrophils [19] thus, suggesting a possibility that free ISG15 may stimulate chemotaxis and migration of NK cells into tumors. Strikingly, defective uterian NK cell migration and distribution contributes to embryonic mortality in ISG15 knockout mice [39]. These results further suggest a role of free ISG15 in facilitating NK cell migration, in this case to the embryonic transplantation site [39]. Whether ISG15-dependent NK cell infiltration observed in the current study is causally responsible for tumor regression in nude mice is not known. However, our results that ISG15 induces NK cell infiltration and tumor regression, and literature reports that NK cells have the ability to kill tumor cells [40] and free ISG15 activates NK cells in culture [18], suggest such a possibility. Our results also explain why NK-dependent tumor regression remained unaffected in ISG15 knockout mice in a previously published study [41].

Decreased MHC-class I expression on tumor cells lead to NK cell activation [42]. In contrast, our current studies have revealed that intracellular free ISG15 enhances MHC-class I surface expression. These contradictory results could be explained by the plausible distinct functions of the two forms of free ISG15 (extracellular and intracellular) *in vivo*. Extracellular free ISG15 is an immune cytokine [18], and a chemotactic agent [19], whereas intracellular free ISG15 has been identified as a potential tumor antigen [22]. It is possible that free ISG15 may function differently in the different parts of the cell. When secreted into the extracellular milieu it may function to establish antitumor innate immune response by activating/proliferating NK cells, and/or by inducing chemotaxis of the activated NK cells towards tumors *in vivo*. On the other hand, intracellular free ISG15 may contribute to activate the adaptive arm of the immune system by enhancing MHC-class I antigen presentation *in vivo*. Both extracellular and intracellular ISG15 together may possibly function to induce robust activation of the antitumor immune response *in vivo*. Consistent with the model, free ISG15 inhibits tumor growth *in vivo* (current study), and vaccination against free ISG15 results in CD8-mediated reductions in both primary and metastatic mammary tumor burden in mice [22]. Further studies are needed to determine whether free ISG15 indeed has potential to induce the adaptive and innate arms of the immune system *in vivo*.

Similar to our cancer-related study, several other literature studies have indicated that free ISG15 may also function to help the immune system to recognize pathogen (viruses and bacteria) infected cells for their destruction in the human body [5, 43]. For example, recent studies by Bogunovic *et al.* has revealed that ISG15 is essential for the secretion of IFNβ from NK cells, consequently establishing IFNβ-mediated anti-mycobacterial immunity in human MSMD (Mendelian Susceptibility to Mycobacterial Disease) patients [44]. Based on this report and our current study, we hypothesize that free ISG15 protein, which is designed to help the immune system to protect the body against viruses and bacteria, may also aid immune cells in detecting cancer cells for their elimination in the human body. Tumor cells deregulate the function of free ISG15, probably by blocking its secretion by conjugating it to cellular proteins consequently, escaping immune surveillance.

Two related studies recently have demonstrated that ISG15 promotes tumorigenesis in mice [45, 46]. In both these papers authors have used ISG15 siRNA to silence ISG15 expression to define the role of ISG15 in tumorigenesis. Since ISG15 siRNA can silence both free ISG15 as well as conjugated form of ISG15, it remains unclear whether ISG15 siRNA-mediated suppression of tumor growth observed in these studies is causally related to the suppression of free ISG15 and/or ISG15 conjugates. Notably, Huang *et al*. study has revealed that ISG15 deficiency suppresses Ki-Ras-driven lung tumorigenesis, and ISGylation of p53 may contribute to tumorigenesis *in vivo* [46]. This data together with our study that ISGylation governs oncogenic function of Ki-Ras [14] suggest that ISGylation may promote tumorigenesis *in vivo*. Interestingly, Ki-Ras-transformed cells were found to be sensitive to lysis by NK cells by other investigators [47]. Plausible involvement of the ISG15 pathway (free ISG15 and/or ISG15 conjugates) in NK-cell-mediated killing of Ki-Ras transformed cells, if any, need further exploration.

We conclude that free ISG15 has a potential to induce an antitumor immune response *in vivo*, a function of free ISG15 that was overlooked in all cell culture *in vitro* and *in vivo* studies published so far. These results together with other empirical evidence that ISGylation has protumor function [13-15] suggest that increased ISGylation and decreased secretion of free ISG15 may lead to cancer. Indeed, expression of free ISG15 and ISG15 conjugates is heterogeneous in human solid tumors and tumor cell lineages [12]. These results thus provide the basis for developing strategies to decrease ISGylation and increase systemic levels of free ISG15 to treat cancer patients overexpressing the ISG15 pathway (*e.g.* pancreatic tumors).

## MATERIALs AND METHODS

### Cells

Human MDA-MB-231, ZR-75-1, and T47D breast cancer cells were obtained from the American Type Culture Collection (Manassas, VA, USA). ZR-75-1 and MDA-MB-231 cells stably expressing control, ISG15 and UbcH8 shRNAs are described in [13, 14]. All cells were cultured in RPMI media supplemented with 10% fetal bovine serum, L-glutamine (2 mmol/L), penicillin (100 units/ml), and streptomycin (100 μg/ml). Breast cancer ZR-75-1 stable transfectants were maintained in hygromycin B (100 μg/ml), and MDA-MB-231 stable transfectants were maintained in puromycin (50 μg/ml). All cells were maintained at 37^0^C in a 5% CO_2_ incubator.

### Immunoblotting

Cells were lysed in lysis buffer containing 50 mM Tris-HCl, pH 7.5, 2% SDS, and protease inhibitor cocktail. Lysates were sonicated, boiled, and cleared by centrifugation. Lysates containing equal protein were then mixed with 6X Laemmli SDS sample buffer (3x final concentration). Samples were boiled again and proteins were separated by 15% SDS-PAGE. Immunoblotting analysis was carried out using anti-ISG15 antibodies, and enhanced chemiluminescence (ECL) Western procedure (Pierce) [13, 14]. The signals were detected using the BioRad VersaDoc Imaging System (BioRad).

### Flow cytometry

Cells (1 × 10^6^) were placed into 5 ml Falcon tube and washed with phosphate-buffered saline. Cells were then stained with anti-HLA class I ABC-PE antibody (eBioscience) for 20 min or mouse IgG2a K isotype control-PE antibody (eBioscience) for 20 minutes at 4^0^C. Cells were then washed with phosphate-buffered saline, and fixed with 1% paraformaldehyde. Flow cytometry was performed using BD FACS flow cytometer (BD Bioscience). BD FACSDiva (BD Bioscience) software was used to analyze results.

### Acid stripping and flow cytometry

Cells (1 × 10^6^/point) were centrifuged in 15 ml conical tube. The resulting pellet was re-suspended in 50 μl of acid buffer containing 300 mM glycin and 1% BSA in water, pH 2.4 [30]. Cells were incubated for 90 seconds at 37^0^C. To neutralize acid, 15 ml of complete RPMI medium was immediately added to the tubes containing cells. Cells were centrifuged and washed with 15 mls of RPMI medium three times (1000 rpm x 5 min). Cells were then replated in 35 mm culture dishes and incubated in the presence/absence of MG132 (5 μM) for six hrs in 37^0^C CO_2_ incubator. Cells were then collected by brief centrifugation, washed, stained using anti-HLA class I ABC-PE antibody, fixed, and analyzed by flow cytometry as described above.

### Tumor studies

*Using ZR-75-1 breast cancer cells:* Six week-old female athymic NCr^nu/nu^ mice (Jackson Laboratory) were anesthetized with Ketamine (100 mg/kg) and implanted subcutaneously with an estrogen-release pellet containing 17β-estradiol (0.72 mg/pellet 60d release) (Innovative Research of America, FL). Two days post implantation, ER positive ZR/control and ISG15 shRNA cells (2 × 10^6^ cells) were subcutaneously injected into the right flank of nude mice (n=8; four mice/group in two different experiments). Tumor dimension was measured using calipers two weeks after the implantation of breast cancer cells.

*Using MDA-MB-231 breast cancer cells:* ER negative MDA/LV-control (Group I) and ISG15 (Group II) shRNA cells (2 × 10^6^ cells) were subcutaneously injected into the flanks on both sides of Ncr^nu/nu^ mice (Taconic Laoratory) (n=4/group; total eight tumors). In Group III, MDA/LV-control shRNA cells (2 × 10^6^ cells) were subcutaneously injected into one flank of Ncr^nu/nu^ mice (n=4/group; total four tumors). On the same day, in Group III mice, recombinant free ISG15 (188 ug) (Boston Biochemical) was injected near the site of xenograft implantation. We tested endotoxin contamination in the commercial ISG15 preparation using ToxinSensor^TM^ Chromogenic LAL Endotoxin Assay Kit (GenScript). The endotoxin level in ISG15 sample was determined from the endotoxin standard curve generated by Chromogenic Assay described by manufacturer. Tolerated Endotoxin limit for a mouse was calculated using the formula K/M, where K is the threshold human pyrogenic dose of endotoxin per/kg body weight [5 EU/ml, United States Pharmacopeia (USP)-approved dose], and M is equal to maximum recommended human daily dose of drug per kg of body weight, as described in [48]. We found that a single dose of ISG15 injected into mice in our experiment had 28 EU/ml, which is below the tolerated endotoxin level (36 EU/ml) judged acceptable for *in vivo* mouse studies.

Tumor dimension was measured using calipers over a three week period. Tumor volumes were calculated using the modified ellipsoid formula (1/2(length x width^2^). Statistical analysis of any changes in tumor volume was carried out using a standard Student's two tailed t test. Animal study was approved by the LSUHSC-NO Institutional Animal Care and Use Committee under its assurance (# A3094-0) with the Office of Laboratory Animal Welfare of the National Institutes of Health.

### Immunohistochemistry

Frozen tumor tissue sections (5 μm) were fixed in zinc-buffered formalin (Z-fix) for 15 min. Tissue sections were permeabilized and dehydraded in −l20°C acetone for 5 min. Endogenous peroxidase was quenched with 3% hydrogen peroxide in PBS for 10 min. After washing with water, sections were incubated with protein block (Dako), and then with either anti-NK cell-specific anti-CD49b-DX5 clone (for ZR-75-1) or -HMα2 clone (MDA-MB-231) (eBioscience) or anti ISG15 antibodies overnight at 4°C. Sections were then incubated with anti-rabbit secondary antibody (Vector laboratory) (for ZR-75-1), and anti-hamster-biotin (Invitrogen) (for MDA-MB-231) for 45 min. After washing with PBS, slides were incubated with Streptavidin-HRP (Dako) (1:500) for 30 min, followed by Diaminobenzidine (DAB) substrate for 5 min. Slides were finally washed with distilled water, dehydrated in ethanol series, cleared in xylene, and covered with Permount. Sections were photographed under Nikon E600 fluorescent microscope (Nikon Instruments Inc.) with 40X (for ZR-75-1) and 20X (for MDA-MB-231) magnification.
